# Towards a Safer Internet of Things—A Survey of IoT Vulnerability Data Sources

**DOI:** 10.3390/s20215969

**Published:** 2020-10-22

**Authors:** Marcin Rytel, Anna Felkner, Marek Janiszewski

**Affiliations:** Research and Academic Computer Network (NASK), Kolska 12, 01-045 Warsaw, Poland; afelkner@nask.pl (A.F.); marekj@nask.pl (M.J.)

**Keywords:** Internet of Things, network security, databases, computer security, data security

## Abstract

The security of the Internet of Things (IoT) is an important yet often overlooked subject. Specifically, the publicly available information sources about vulnerabilities affecting the connected devices are unsatisfactory. Our research shows that, while the information is available on the Internet, there is no single service offering data focused on the IoT in existence. The national vulnerability databases contain some IoT related entries, but they lack mechanisms to distinguish them from the remaining vulnerabilities. Moreover, information about many vulnerabilities affecting the IoT world never reaches these databases but can still be found scattered over the Internet. This review summarizes our effort at identifying and evaluating publicly available sources of information about vulnerabilities, focusing on their usefulness in the scope of IoT. The results of our search show that there is not yet a single satisfactory source covering vulnerabilities affecting IoT devices and software available.

## 1. Introduction

The Internet of Things (IoT) is a concept of connected smart items that keeps gaining increasing popularity and is already becoming a reality. However, as in case of many innovations, the security aspects related to the IoT are not always well recognized and established. Very symptomatic is the fact that there is no strict, widely accepted definition of the IoT term itself. Because of that, for the purpose of this work we define an IoT device as any item equipped with network connectivity and the ability to collect and exchange data, except a phone, PC, tablet and data centre hardware. By this definition IoT includes devices such as smart home appliances, routers, intelligent cars, closed-circuit television (CCTV) cameras, wireless sensors, and a wide range of industrial control systems (ICS) and industrial Internet of Things (IIoT) devices, which are often regarded as separate from the IoT. The excluded devices are personal computers, tablets, mobile phones etc., as well as software designed to run on them—unless it is directly related with some IoT device, such as applications used to control it or read its status. While smartphones are sometimes considered to be IoT devices, we decided to exclude them from our definition. Their versatility and constantly growing computing capabilities led us to classify them as mobile computers rather than simple internet connected “things”.

**Figure 1:**
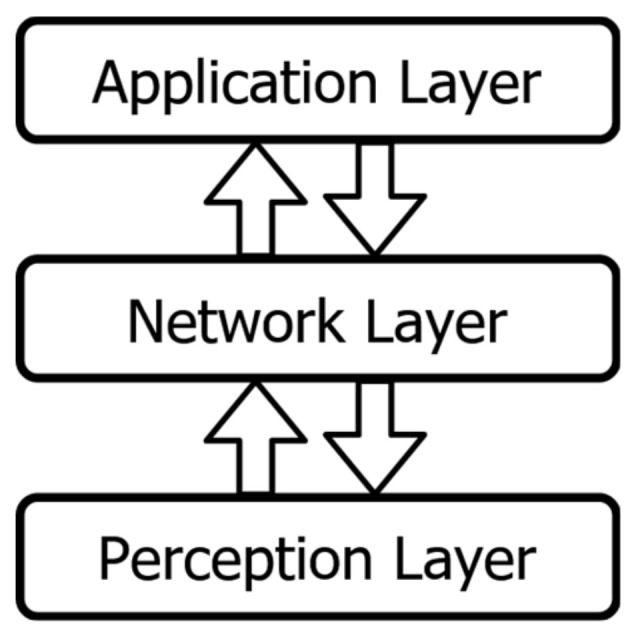
The common three-layer IoT architecture.

The IoT paradigm is broader than just the devices, as it also includes the connections between devices, servers and users, as well as data collection and processing. It can be broken into three distinctive layers [1], as presented in Figure 1.

These basic IoT layers are:

1. Perception layer: The physical devices themselves, e.g., sensors, actuators, smart things, etc.
2. Network layer: The communication infrastructure for the devices, servers, and users.
3. Application layer: The software that can use the data obtained from the devices and can manage them, providing services for the end user.

Some works propose more layers. For example, Dorsemaine et al. [2] adds a separate layer for data storage and data mining, and notes that only the first layer is specific to the IoT. Furthermore, [3] presents three consecutive layered IoT architectures, each adding an additional layer, up to the latest five-layer architecture.

The current state of the IoT security is alarming. While the awareness of security aspects of traditional IT is at a relatively high level, with well-defined procedures and rules, the security in IoT—which comes with its own unique challenges—remains in its infancy. Many manufacturers do not have prior experience with cyber security and often neglect it. To illustrate the problem, the IoT Security Foundation research conducted in 2018 [4] showed that only 10% out of 331 IoT companies sampled had any vulnerability disclosure policy in place. Multiple vendors ignore even severe issues found within their products, as seen in the case of GPS trackers leaking sensitive information [5], even though their primary use case is to aid the security of the user. Furthermore, even the vulnerabilities found in IoT devices, disclosed to the vendors, and hopefully mitigated by them are rarely disclosed to the public by the vendor itself. For example, the Chinese brand Xiaomi, which offers multiple IoT products and has its own active bug bounty program [6], does not announce the found and mitigated vulnerabilities, resulting in a very low amount of publicly known vulnerabilities. At the time of this writing there are only 43 found in the well-known American National Vulnerability Database (NVD) [7] affecting this vendor, most of which concern smartphones. Therefore, while the total amount of vulnerabilities is supposedly large, there is no universal way to track and mitigate them. The immaturity of IoT security is reflected in the most common threats listed in the 2018 Open Web Application Security Project (OWASP) Top 10 Internet of Things list [8], with the top threat being “weak, guessable, or hardcoded passwords”, closely followed by lacks in encryption, authorization, and updating. The lack of update mechanisms in IoT devices is especially dangerous, as it hinders the vendors’ ability to remotely mitigate the well-known vulnerabilities. Oftentimes the vulnerabilities are found within devices’ hardware and can only be fixed by modifying the hardware itself, which is costly and hence it rarely happens. This weak state of IoT security has been already exploited multiple times, most famously by the Mirai botnet performing large scale distributed denial-of-service (DDoS) attacks [9]. Other forms of device abuse let an attacker mine cryptocurrencies, steal victims’ private data, disturb functioning of smart houses and smart cities or, in the case of IIoT and ICS, cause physical damage to the industry or infrastructure [10].

The IoT world differs significantly from the traditional IT world. Information about vulnerabilities of IoT devices should reflect the IoT paradigm and contain information which layer of IoT architecture the vulnerability affects as well as specific information about the device itself, such as protocols used, physical specification, etc. Most currently accessible information on IoT vulnerabilities does not contain such data and it cannot be easily obtained. A comprehensive IoT vulnerabilities database should enable the storage of such information, but we have to accept that in the beginning we do not get such information for many vulnerabilities.

The knowledge about vulnerabilities affecting the device is essential to mitigate them. The most desirable source would be a database covering the IoT vulnerabilities. A small scale attempt in creating such database was done by the University of Central Florida [11], but the database is no longer accessible and its data set was not publicly available. This leaves an open space for a new IoT oriented vulnerability database. Creating such a database is one of the long-term goals of our work.

The most popular source of vulnerability information is the NVD, which is synchronized with the CVE (Common Vulnerabilities and Exposures) list and describes only vulnerabilities with the CVE entries assigned. Most of the general vulnerability databases lack any built-in categorization, which would help pick out vulnerabilities regarding IoT devices from their datasets. Thus, using these databases as a primary source for automated information gathering requires prior knowledge about which assets belong to the IoT world. The smart device market is diverse and rapidly growing with an abundance of original equipment manufacturers (OEM), whose products are marketed by different companies under multiple trade names, making this requirement hard to satisfy.

The article can be perceived as the first, small, but crucial step to create a comprehensive IoT vulnerabilities database. As we do not provide any technical recipe for such a database in this paper, we have identified the most important sources of information that should be taken into consideration and we evaluate them. The following sections summarize the results of our research in identifying and evaluating potential data sources about vulnerabilities affecting the Internet of Things.

The paper is organized as follows. Section 2 explores related articles, mostly focused on IoT vulnerabilities and vulnerability databases. Section 3 contains description of each source identified by us. It is followed by Section 4, which compares the most relevant sources. Section 5 summarizes our findings, and Section 6 discusses our plans for future work based on findings described in this paper.

## 2. Related Works

We found a similar work that explores possibilities of obtaining IoT vulnerability data from publicly available sources using data crawling [12]. However, it is focused on data acquisition and presentation rather than the sources themselves, as it only uses three different sources. Rizvi et al. [13] propose a security taxonomy for IoT, and [2] proposes a general IoT taxonomy. Security and privacy requirements for IoT are presented in [14], and this work provides a real-life example by exploiting an IP camera system. The work about vulnerable IoT devices in Jordan [15] explores devices affected by selected well-known vulnerabilities found in the CVE List. A similar study [16] uses Nessus vulnerability scanner to find vulnerabilities in consumer IoT devices representing three categories: smart TVs, webcams and printers. Miettinen et al. [17] use fingerprinting and machine learning to identify IoT devices within a network and use CVE data to assess their vulnerabilities. A survey on IoT vulnerabilities [1] presents multiple articles discussing IoT vulnerabilities, but their scale is limited. None of them tries to approach the issue of cataloguing all known vulnerabilities. Another recent work on IoT vulnerabilities [18] does not mention any dedicated source of known IoT vulnerabilities either. There is, however, a catalogue of sources for general vulnerability data, namely the Vulnerability Database Catalog on the Forum of Incident Response and Security Teams (FIRST) website [19]. Finally, [20] notes that currently available databases lack information about vulnerabilities found in low-level components, which would be beneficial in the IoT context.

## 3. Data Sources Overview

This section contains descriptions of the sources that we considered in our research. Each of the main sources, containing a significant number of entries or particularly interesting from the IoT standpoint, is described in its separate subsection. Minor sources, containing fewer entries or difficult to quantify, are grouped into larger categories and characterized collectively. The sources described in this section are briefly characterized in Table 1.

### 3.1. NVD

NVD [7] is a general vulnerability database maintained by the American National Institute of Standards and Technology (NIST) [21]. It analyses and scores vulnerabilities that have their unique CVE [22] identifier granted. The identifier consists of three parts separated by hyphens:

- “CVE”
- Year (4 digits)
- Vulnerability number (4 or more digits)

Example: CVE-2010-1677.

NVD uses two scoring systems: Common Vulnerability Scoring System (CVSS) v2 [23] for all vulnerabilities, and, since late 2015, CVSS v3 [24]. While widely used and broad in scope, this database was created with focus on the software vulnerabilities. Hence, it is lacking as a primary source of information about IoT vulnerabilities, which usually apply to the specific devices. It has no categorization, which would allow distinguishing IoT from other products. The closest substitute lies within the common platform enumeration (CPE) [25] identifiers used to unambiguously indicate the vulnerable product, specifically in the “part” field, which can contain either “o” (operating system), “a” (application) or “h” (hardware). However, the scope of the “h” part is much broader than the IoT itself, as it also includes hardware products such as microprocessors or mobile phones. Moreover, many IoT vulnerabilities are found outside of the hardware, but also in its OS or applications. These applications associated with the specific device can run on hubs, mobile phones, computers, or remote servers. Further difficulties with using CPE arise due to the common practice among IoT vendors, marketing the same devices produced by the OEM manufacturers under many different vendor brands and product names.

NVD provides data in two ways, directly on its website and in a regularly updated JSON feed. The information available on the website is slightly richer than that found in the feed, which lacks data such as revision history or CVSS scores assigned by the CNAs (CVE Numbering Authorities). The data fields available from this source are summed up in Table 2.

The complete specification of the JSON data feed can be found in [26]. Some NVD vulnerabilities have an annotation stating that data regarding the vulnerability has changed since the last entry update. This information is unavailable in the JSON feed. Notably, unlike most other sources, NVD does not give any titles to its vulnerability entries. Another notable difference from many other sources is that NVD entries do not have a dedicated field for vulnerability mitigation information, separate from the general references field.

### 3.2. CNVD

Another general vulnerability database is the China National Vulnerability Database (CNVD) [27] maintained by the Chinese national CERT—National Computer Network Emergency Response Technical Team/Coordination Center of China (CNCERT/CC) [28]. This database uses its own vulnerability identifier, similar to the CVE ID. It contains three parts, separated by hyphens:

- “CNVD”
- Year (4 digits)
- Vulnerability number (4 digits up to 2012, 5 digits from 2013 onwards, left-padded with zeroes)

Example: CNVD-2018-10443.

Besides the usual list containing all disclosed vulnerabilities, CNVD separates vulnerabilities based on the type of software or device that they concern. Currently, there are 3 subpages and 8 categories, as presented in Table 3. The categories are selectable within the CNVD website and are presented in the same way as the general vulnerability list. Subpages use different page layout and are presented on separate sites, each using its own subdomain (telecom.cnvd.org.cn for Telecommunications, mi.cnvd.org.cn for Mobile Internet and ics.cnvd.org.cn for ICS). However, vulnerability URLs listed on these subpages link back to the main CNVD domain (i.e., www.cnvd.org.cn). The category or subpage of a given vulnerability is only visible from the specific subpage or category list. In other words, it cannot be simply found on the vulnerability description page itself. Categories relevant in the scope of IoT are smart devices, network equipment, and ICS. The mobile internet and telecommunications subpages are not monitored despite their supposed relevance. The first one focuses on mobile phones and lists mainly vulnerabilities found in Google Android OS and Apple iOS. The latter includes both vulnerabilities found in customer-grade (e.g., home routers and switches) and industry-grade network devices (e.g., CISCO core network devices and operating systems). While the first group falls within our interest, most of its entries are also included in the network equipment category and are hereby already observed. The sum of vulnerability counts across all categories is smaller than the total vulnerability count, implying that there are also uncategorized entries. No overlap was noticed between the two observed categories (smart devices and network equipment), but there is an overlap between categories and subpages i.e., vulnerability listed under the ICS subpage can also be present in the IoT device category, network device category, or neither of them.

Beside the categories listed above, the CNVD has created lists for one more blockchain related category and an internet of vehicles category, but these are currently empty. The subpages also list links to the blockchain subpage which, unlike the other three subpages that include only vulnerability lists, is a functional sub-portal within the CNVD.

CNVD provides XML vulnerability feed for its registered users. The feed is updated weekly, each Monday at 18:00 CST (UTC+08:00) with a new file containing vulnerabilities published during the past week. The XML feed offers only partial data, missing fields containing vendor solution, CVSS score, external IDs other than CVE and Bugtraq, database inclusion date and update date. It is also impossible to determine the category based only on the feed information. Moreover, the XML data are not modified when the vulnerabilities that it contains receive updates. Another downside of this feed are missing files. There are a few weeks without their corresponding XML files available. The vulnerabilities published during the missing weeks are not included in later XMLs either, making them inaccessible using the feed alone. The feed was started in January 2015 and vulnerabilities published earlier were not included in its files. The data fields available on the website and in XML files are summarized in Table 4.

**Figure 2:**
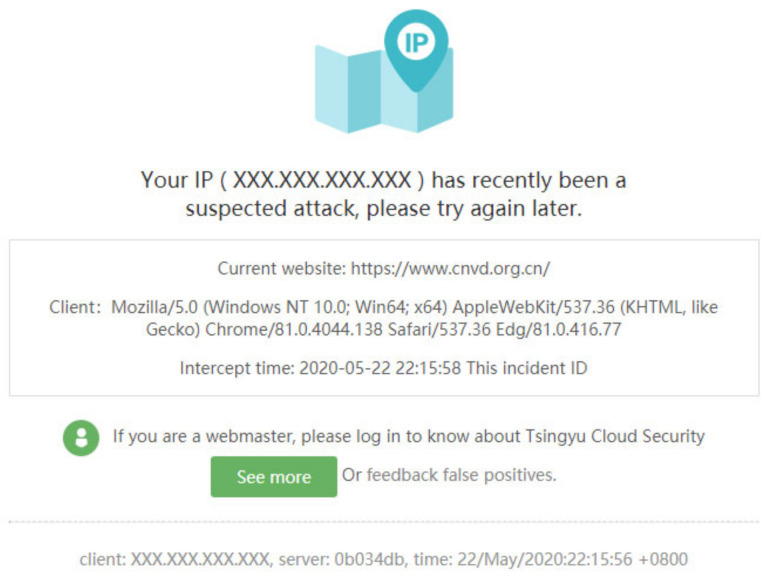
Example of CNVD access restriction message, anonymised screenshot of [27] accessed during the restriction period.

The main drawback of CNVD is that it does not provide any straightforward way to download its entire database. There is no API, and the available data feed is incomplete. Moreover, the traffic rate to the website is being limited. The access from a given IP address may be temporarily restricted if the request frequency is too high. This restriction usually lasts 90 s and can be worked around by using a different IP address. When the access restriction occurs, one of the error screens is presented instead of the desired page. There are a few similar messages that can be displayed to the user, and one of them is presented in Figure 2.

Most of the CNVD vulnerability entries contain references to the external sources and external vulnerability IDs, notably CVE [22] and Bugtraq [29] ID. The majority of entries contain a single CVE or no CVE reference at all, with rare exceptions. The observed CVE counts are presented in Table 5.

The number of entries analysed here—11,421—Covers 7.8% of the total CNVD vulnerability count (i.e., 146,342 on 25 August 2020). The vulnerabilities included in the table originate from the IoT-related categories.

The CNVD vulnerability database features some errors. For example, there are two different vulnerabilities listed under the same ID—CNVD-2017-37032. Since the vulnerability description URL is determined by its ID, only one of these vulnerabilities is actually available via the web service. The shadowed vulnerability can still be accessed in the XML feed. Another issue is the fact that the data displayed on the website sometimes contain non-escaped special characters in some fields, resulting in corrupted webpage rendering.

The CNVD scores the vulnerabilities using CVSS v2 [23]. The vectors frequently differ from these provided by the NVD for the same CVEs, and there are numerous vulnerabilities with scores provided that do not contain CVE ID, implying that the scoring—at least in some cases—is done by the database maintainers themselves. The CVSS scores comparison between CNVD and NVD is presented in Table 6.

Out of all 11,421 CNVD entries checked, 8169 (71.5%) had both CVE ID and CVSS vector, allowing us to compare the vectors between CNVD and NVD, so the percentages in Table 6 are calculated in reference to this value. Among these vulnerabilities, only 59.1% had exactly the same CVSS v2 vectors in both databases, 40.1% entries had one or more differing metrics, and 0.5% of CVEs referenced by the CNVD were not actually found in the NVD. A few of them were checked manually and turned out to be either deleted duplicates or CVE candidates. Finally, 0.3% entries were present in the NVD but were missing CVSS v2 scores there. These were new vulnerabilities that are still awaiting analysis by NIST.

### 3.3. CNNVD

Chinese National Vulnerability Database of Information Security (CNNVD) [30] is a vulnerability database maintained by the Chinese government agency, China Information Technology Security Evaluation Center (CNITSEC) [31]. The database introduces its unique vulnerability identifier, consisting of 3 parts separated by hyphens:

- “CNNVD”
- Year (4 digits) + month (2 digits)—6 digits
- Vulnerability number (3 or more digits, left padded with zeroes).

Example: CNNVD-202001-005.

The data provided by this database may be manipulated by the Chinese government, as discussed in [32]. Unlike CNVD, CNNVD has no vulnerability categorization. The entire database is available for download in XML files. As in NVD, vulnerabilities from each year are put in a single file with two additional files, one containing vulnerabilities from the current month and a second one containing the vulnerabilities added or updated on the current day. However, CNNVD limits the XML files availability only to the registered users. Unfortunately, the registration ability is strictly limited. Hence, the preferred way of obtaining the data is web scraping. As CNNVD does not limit requests in any noticeable way, the entire database can be scraped in a reasonable time. The data fields describing vulnerabilities are presented in Table 7. As the XML feed is not publicly available, only data from the website are shown.

**Figure 3:**
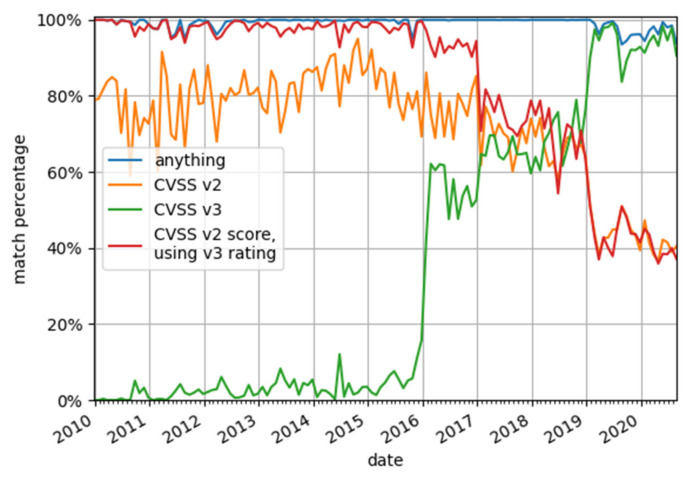
CNNVD severities matched with severities based on the NVD CVSS scores. Orange line—Matching with CVSS v2 rating. Green line—Matching with CVSS v3 rating Red line—Matching with rating based on CVSS v2 score but using CVSS v3 severity ratings. Blue line—Matching with any of the previous ratings.

CNNVD usually follows NVD. The majority of its entries (141,109 out of 149,580, i.e., 94.3%) provide CVE ID. CNNVD does not provide CVSS vectors of scores, with the only available rating being the severity. The basis used to determine the severity rating is not explicitly stated. Out of 8471 vulnerabilities without CVE ID, only 1824 (21.5%) have a severity rating provided, but the ratio becomes as high as 99.5% (140,406 out of 141,109) for vulnerabilities with CVE identifiers. For vulnerabilities with CVE IDs, the severities displayed by CNNVD were compared to the severities evaluated by the NVD. The values do not match perfectly. Most noticeably, many vulnerabilities with severity rating high in NVD are rated critical by CNNVD. This suggests that CNNVD uses NVD’s CVSS score to determine the severity rating but using different criteria. Indeed, using NVD CVSS v2 scores with v3 severity ratings, described in [33], results in a close match with CNNVD severities for vulnerabilities published before 2017. Since then, the severities based on CVSS v3 scores are being used in parallel to the ones based on CVSS v2. Between 2016 and 2019 there is no consistence in the ratings used—The CVSS version is seemingly picked at random by the Chinese editors. Starting in 2019, the CVSS v3 takes precedence and becomes a dominating scoring system. The graph presenting the compliance of CNNVD severities with NVD scores is shown in Figure 3. The graph includes four lines, one for each scoring system and one depicting compliance with any of the three systems. The data is grouped by months (i.e., each data point represents one month). The month is determined by the CNNVD ID, e.g., entries with IDs starting with CNNVD-201906 are counted in June 2019.

The severity ratings for given CVSS scores are presented in Table 8.

A similar analysis performed in February 2020 was showing better matches in years 2010–2013 than the current one, done in August 2020. We speculate that the difference is due to entry updates in the NVD database—Scores for older vulnerabilities are sometimes re-evaluated in NVD, but the corresponding CNNVD entries are not updated after this re-evaluation.

CNNVD uses CPE identifiers internally and shares them in their XML feed, but they are not fully available on the vulnerability description page. Specifically, the affected products list displayed on the page contains partial CPE data (vendor and product name, version number) with stylistic change, usually capitalized names and a separator between vendor and product changed from a colon to a whitespace. Exemplary entry: Google Android:4.2.

### 3.4. JVNDB

Japan Vulnerability Notes iPedia (JVNDB) [34] is a vulnerability database maintained by the JPCERT Coordination Center and Information-technology Promotion Agency (IPA) from Japan [35]. The database entries are written in Japanese, but a subset of its data is also available in English. JVNDB uses its own identifiers:

- “JVNDB”
- Year (4 digits)
- Vulnerability number (6 digits, left padded with zeroes)

Example: JVNDB-2016-000214.

Similarly, to CNNVD, most data in JVNDB is closely related with NVD, with relevant CVE identifiers provided within JVNDB entries. The total number of entries in these databases is comparable. NVD and CNNVD are approaching 150,000 while JVNDB exceeds 120,000 at the time of this writing. Nevertheless, JVNDB contains some unique vulnerabilities that are not covered elsewhere, often regarding products originating from Japan. Additionally, some vulnerabilities found in both NVD and JVNDB are analysed independently by IPA, providing another view onto them, sometimes different than given by the NVD. The database contents are publicly available through an XML feed, but the data it provides is incomplete, so to retrieve all the information it has to be supplemented by scraping data from the website. For example, some entries can contain multiple CVE IDs and CVSS scores, but the feed contains scores for only one CVE. The data fields available in the database entries are presented in Table 9.

Outside of Japan, the JVNDB may be redundant as the primary source of vulnerability information, since most of its entries are overlapping NVD. However, it is still useful as an additional source, as some vulnerabilities are unavailable in other sources and other entries can provide more insight into previously known ones.

### 3.5. IVD

**Figure 4:**
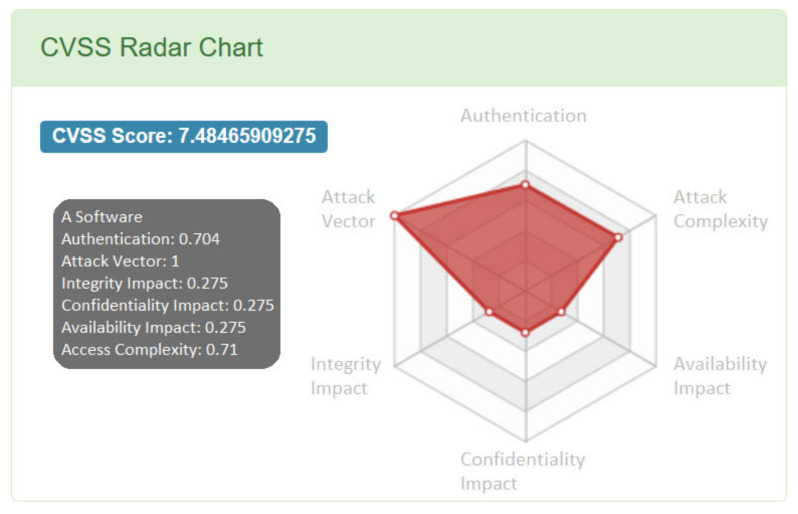
Example of IVD CVSS score radar, reproduced from [37].

The ICS Vulnerability Database (IVD) [36], maintained by Chinese company Winicssec Technologies, is focused solely on vulnerabilities found in the industrial control systems. The majority of its entries are based on information from NVD, CNVD and CNNVD, often tying all three identifiers together. The vulnerabilities without external IDs are a minority and present little value due to their limited descriptions and affected products listings. The database does not introduce its own identifier other than a random hex number used in the vulnerability description page URL. For almost a year, between January 2019 and January 2020, the database was considered no longer maintained, since no new vulnerabilities were being added. This has changed on 21 January 2020, when substantial changes to the database’s content were observed: 757 new entries with vulnerabilities from 2019 and early 2020 were added, and 1157 old entries were deleted, though some of these changes were entry updates with subsequent ID change. The IVD uses CVSS v2 but presents the ratings in an unorthodox way. Instead of a vector, the numerical values representing each metric are displayed on a visual “radar”, as shown in Figure 4. The overall score displayed on the website does not comply with the CVSS v2 standard as it is not properly rounded to the first decimal.

For each metric the values displayed in this example match these specified by the current CVSS v2 equations [38], which lets one reconstruct the CVSS vector from them (AV:N/AC:L/Au:N/C:P/I:P/A:P for the radar data shown above). This is not the case for some entries, as it was observed that the values shown on the radar are sometimes derived from older versions of CVSS v2 specification, which can be found in [39]. Moreover, there are entries with values impossible to match with any specification or missing some metrics entirely. The compliance with the latest CVSS v2 specification had arisen after the database update in January 2020. The amount of non-compliant entries dropped from 1062 out of 3538 (30.0%) before the update to 229 out of 3136 (7.3%) after. Most of these 229 entries left have missing scores in every metric. The vulnerability data available in each entry is usually the same as data available in the referenced Chinese database, particularly CNVD. The data fields are presented in five groups:

- Vulnerability parameters: basic vulnerability information, including its type, IDs in other databases and publication date.
- CVSS radar chart.
- Affected platforms and products.
- Vulnerability description.
- Security suggestions and solutions.

As IVD offers little knowledge that cannot be found in other sources, it is most useful as an additional source to help categorize the known vulnerabilities as affecting ICS.

### 3.6. ICS-CERT-CN

Chinese ICS CERT is a part of CNCERT/CN, the caretaker of the CNVD database described in Section 3.2. It hosts its own vulnerability list focused mainly on the ICS vulnerabilities [40]. 3138 vulnerability entries were checked. Each entry receives its own identifier, but the vulnerability descriptions are all copied either from either CNVD (2858, or 91%) or CNNVD (259, or 8%). The remaining 21 vulnerabilities are visible on the vulnerability list, but their description pages are unreachable. The data is hosted as a local mirror of the entries, as some CNVD vulnerability entries referenced by the ICS-CERT are removed from the CNVD, but are still available in the ICS-CERT. Entries originating from both Chinese databases have the same field labels, which leads to a minor inaccuracy—CNNVD IDs are presented in a field labelled “CNVD number”. While this list does not provide any vulnerabilities not already present in the Chinese databases, it helps to categorize them. There are two categories, namely “industrial control” (2662 entries, 85%) and “Internet of things” (476 entries, 15%). Some entries from these categories were not found in their respective categories in the CNVD or the IVD database (283 in the ICS category and 57 in the IoT category). A further 202 CNVD IDs were marked as related in the ICS-CERT entries, resulting in a total of 542 additional CNVD vulnerabilities that were not previously identified as IoT or ICS related. Whilst for most entries there is no data added over the source database, there are some exceptions. For example, a few entries contain a map of China roughly showing locations of vulnerable devices. It is unknown whether these data are accurate and up to date, but their utility is low anyway since they only includes devices located in China, and the location precision is limited to pinpointing the province. All entries containing the map were published in 2018, which suggests that this feature was scrapped after a short trial. Similarly, to IVD, the Chinese ICS-CERT’s vulnerability list is mostly useful to categorize the referenced CNVD and CNNVD entries as ICS or IoT related. Another similarity to IVD is an infrequent updating of the vulnerability list, which tends to happen only once every few months.

### 3.7. US-CERT

The American CERT [41] issues weekly vulnerability bulletins, with short descriptions and links to other sources, including NVD. This data feed is not particularly useful, as it contains data already available through NVD. The more interesting information is contained in ICS-related advisories. They provide information on vulnerabilities found in ICS devices (IDs starting with “ICSA”) and medical equipment (IDs starting with “ICSMA”). These advisories usually refer to CVE IDs, but contain additional information, such as more detailed descriptions and measures to mitigate the vulnerabilities, unavailable in the NVD database. Moreover, ICS advisories tend to be published earlier than the NVD entries regarding the same vulnerabilities. The advisories are presented in a human-readable format, which is more verbose and harder to process automatically than typical entries from vulnerability databases but includes more detailed information. A typical ICS-CERT advisory consists of the following sections:

1. Executive summary: provides a brief summary of the advisory, including CVSS score of the most severe vulnerability, affected vendor and product type and type of vulnerabilities.
2. Update information: notifies the reader if the advisory is an updated version.
3. Risk evaluation: explanation of possible harm due to successful exploitation.
4. Technical details: The main part of the advisory, contains list of affected products, list of vulnerabilities with their descriptions, CVE IDs, CWEs, CVSS vectors, and scores.
5. Mitigations: methods of mitigating the vulnerabilities, such us vendor patches or possible workarounds.

ICS-CERT is the most valuable of the three ICS-oriented sources, as it often presents original data that is not found in the other sources and, unlike IVD and Chinese ICS-CERT, frequently publishes new advisories and updates the existing ones. The downside of this source is that it is not fit for machine processing. The data are presented in a form more suitable for human reader, and there is no additional data feed. Another difference to previous sources, which may be considered a drawback, is that a single advisory often includes multiple vulnerabilities, while databases usually keep one vulnerability per entry.

### 3.8. Other CERTs

Other CERTs seldom present vulnerability information on their websites. Even if it is presented, vulnerabilities regarding IoT devices are rarely found and lack categorization. The same information is usually also available in NVD, which diminishes the usefulness of these websites even more. However, we identified two additional valuable sources in this category, apart from the already covered US-CERT and ICS-CERT-CN. The first one is Carnegie Mellon University’s Software Engineering Institute CERT/CC [42] which publishes information about newly discovered vulnerabilities, some not found in the NVD. For the vulnerabilities that are eventually included in NVD the publication in CERT/CC usually precedes the NVD. Vulnerabilities are not categorized by the affected products type. The vulnerability notes are available directly on the CERT’s webpage. The data is also shared as JSON files on GitHub [43], but this archive is rarely updated, i.e., once every few months. The second source is the German CERT VDE (Verband der Elektrotechnik, Elektronik und Informationstechnik—Association for Electrical, Electronic & Information Technologies) [44], which focuses strictly on the ICS devices. It provides two data feeds “Advisories”, containing information on vulnerabilities coordinated by CERT@VDE itself, and “Alerts” advisories issued by third parties, namely: US ICS-CERT, Siemens CERT, BOSCH PSIRT and CODESYS.

### 3.9. Zero Day Initiative

Zero Day Initiative (ZDI) [45] is a bug bounty program maintained by the cyber security company Trend Micro Inc. After the discovery ZDI gives vendors up to 120 days to mitigate the issue. After the vulnerability is patched or when the 120 days pass, ZDI publishes advisories regarding the discovered vulnerabilities. ZDI uses two identifiers. The first one is used for publicly disclosed vulnerabilities:

- “ZDI”
- Year (2 last digits)
- Number (3 or more digits)

And the second one, used for vulnerabilities awaiting public disclosure (candidates):

- “ZDI-CAN”
- Number (3 or more digits).
- Examples: ZDI-20-1016, ZDI-CAN-11302.

Most vulnerabilities processed by the ZDI eventually receive their CVE identifiers. CVSS v2 scores and vectors were provided for all vulnerabilities since 2010, including these awaiting public disclosure. Starting in 2019, the scoring system was changed to CVSS v3. There is no product categorization to aid in the selection of the IoT products. The advisories are available only via the website. They are structured similarly to the ICS-CERT advisories, but their contents are usually simpler and each advisory covers only a single vulnerability. The advisories contain the following sections:

- Date of publication
- Title
- ZDI IDs (both regular and ZDI-CAN ID for published advisories)
- CVE ID
- CVSS score and vector
- Affected vendors
- Affected products
- Vulnerability details
- Additional details—This part can contain the exact disclosure timeline, vulnerability mitigation information, and other data.
- Disclosure timeline—Shows most important disclosure dates.
- Credit—Person or organization reporting the vulnerability.

While there is no data feed available and the website presents its data with focus on human readability, it is well structured, simple and consistent among entries, so it can still be automatically processed.

### 3.10. Bugtraq

Bugtraq is a mailing list maintained by Security Focus, with a full disclosure policy. Beside the list itself, it also contains a vulnerability database. Both Security Focus and Bugtraq were acquired by Symantec and, despite prior assurances [46], the database is no longer updated. The website [29] contains numbered vulnerability entries, often referenced by other sources as Bugtraq ID or BID. Each entry includes five tabs:

1. info tab contains basic vulnerability information: Bugtraq ID, vulnerability type, CVE ID, information if the vulnerability is exploitable remotely or locally, dates of publication and of the last update, identity of the vulnerability finder, and a list of vulnerable products.
2. discussion tab contains vulnerability description.
3. exploit tab can include the exploit itself or other information related to exploitation of the vulnerability, such as PoC existence.
4. solution tab describes possible remediations of the vulnerability.
5. references tab shows links to external sources of information about the vulnerability.

Each tab is a separate HTML page. As there is no data feed or API, obtaining full data about a single vulnerability requires visiting all tabs related to it, lengthening the data acquisition process. The source’s usability is currently limited as it is not updated and new entries have not been added since July 2019, but it is still one of the largest sources with over 100,000 entries, and its BIDs are frequently referenced by the sources described in previous sections.

### 3.11. Vulners

Vulners [47] is a service that aggregates cyber security information from multiple sources, ranging from vulnerability and exploit databases (NVD, Exploit DB, Seebug), through vendor security advisories to security-related blogs. Data from 131 sources is currently available, including some of the sources described earlier: NVD, JVNDB, US-CERT and ZDI, and new sources are constantly being added. The data quality and completeness vary. Oftentimes, Vulners data are missing parts of the information found in the original source for individual entries, or not all entries from a given source are available in Vulners. For example, Vulners JVNDB feed includes only entries translated to English by the source, missing these written in Japanese, and Vulners ZDI entries have sometimes missing contents of fields other than description, such as CVSS score and vector or disclosure timeline. The service provides an API that lets one download the data, create its local mirror and keep it up to date. The total number of available entries exceeds 1.8 million, though many of them are the descriptions of the same issues received from different sources. Vulners tries to link entries from different sources together, providing lists of related items with every entry. Unlike other sources described in this paper, Vulners is a paid service, with limited free access to an API for non-commercial purposes and free access to the search engine on the website.

### 3.12. Exploitee.rs

Exploitee.rs [48] is a wiki created by a group dedicated to hacking smart devices. They initially worked only on devices based on the Google TV platform, but later extended their scope of interest to all consumer-grade IoT devices. The entries available on the wiki range from “hacks” that let users add new features to their devices to exploits allowing unauthorized root access. Many entries include pictures from the device’s disassembly, often with universal asynchronous receiver-transmitter (UART) pins locations marked. Entries do not follow any rigid structure and vary from short notes with a single device weakness to long writeups with several well documented exploits. While exploitee.rs should be considered rather an exploit than vulnerability data source, we decided to still include it in this survey as it is the only publicly available source identified by us that focuses strictly on the IoT devices. This is also the only source whose entries can be associated with a single IoT layer, namely the perception layer, since they are all focused on the hardware devices. The website was most active around 2011, and the most recent entry was added in November 2018. In total, entries for 60 devices are available.

### 3.13. Other Structured Sources

There are several other structured sources of information that were considered but are not mentioned above. These include mirrors of the NVD database, which often provide enriched information such as CVEdetails [49], Saucs [50], and HPI-VDB [51], another national vulnerability database, the Russian BDU [52], commercial databases that require payments to freely access their data, VulDB [53], VulnDB [54], security advisories found on websites of various vendors and CERTs, CCTV systems vulnerabilities database [55], listing CVE vulnerabilities affecting CCTV cameras, and other databases that are no longer available. The last category includes closed general databases: Open Source Vulnerability Database (OSVDB) and Chinese Wooyun, three databases focused strictly on IoT, the University of Central Florida IoT database [11], HFDB (Hardware Forensic Database) created by French CERT-DS [56] and secureplanet.io, a start-up whose goal was the creation of open source software vulnerability database focused on IoT, that was cancelled without providing any explanation before the database was even created.

### 3.14. Unstructured Sources

Many IoT vulnerabilities, especially these found in the consumer-grade smart devices, never receive CVE identifiers and are not covered in the vulnerability databases. They can be, however, found on blogs written by researchers that discovered them. The most prominent blog with multiple IoT focused posts is Pen Test Partners [57]. It contains dedicated internet of things and ICS categories with over 150 posts combined, with most of them being descriptions of consumer grade smart devices hacking. Some entries include related CVE IDs, but most of the vulnerabilities seem to lack them. Other security companies’ blogs that also cover IoT, but do not have a dedicated IoT category are, for example: Tenable [58], Attify [59] and Payatu [60]. Other interesting blogs are the following:

- Safegadget, with frequently updated articles containing a list of hacked IoT devices [61].
- Darius Freamon blog [62].
- Embedded Device Hacking blog [63].
- Home of Pierre [64].
- Matthew Garrett’s blog [65].

Beside these listed above, numerous other blogs with some IoT vulnerabilities described can be found over the Internet. The last category of sources includes websites, such as:

- Router Security [66] by Michael Horowitz, a frequently updated source with extensive information about router security.
- RouterPWN [67], an archive of router related information (not updated since 2015, but still active on Twitter).
- Hacking Printers wiki [68], last updated in 2017, created in the Ruhr-Universität Bochum during the work on the survey [69].
- Embedded Device Security subpage of InfoSec Reference [70] a repository of references revolving around hacking embedded devices, including IoT and ICS.

These sources are maintained by individual people, without larger organizations backing them or, in case of the Hacking Printers Wiki, are a by-product of the academic research conducted in a university. As a result, they are rarely supported in the long term and usually stop receiving updates after a few months. Moreover, the data they provide are not structured well, making them even less suitable for use as primary sources in an automated vulnerability management system. Nevertheless, they often contain original research that cannot be found elsewhere.

## 4. Comparison of the Sources

The sources described in the previous section will be compared using different criteria. First, the relevancy of the source in scope of the IoT, favouring either sources with the majority of entries related to the IoT or ones letting one filter IoT vulnerabilities. Next, the individual entries value will be compared, taking into account the use of CVE identifiers, vulnerability scoring systems, and affected product identification.

### 4.1. IoT Relevancy

The sources can be divided into three groups based on the IoT relevancy: IoT focused sources, general sources with some IoT amenities and other sources. The first group includes exploitee.rs, entirely dedicated to consumer-grade connected devices, and three ICS-focused sources: IVD, Chinese ICS-CERT and US-CERT’s ICS section. The second group includes only CNVD, which offers IoT, ICS and Network Devices categories, and serves as a main source for the Chinese ICS-CERT, effectively broadening its ICS and IoT categories by adding vulnerabilities referenced by CERT-CN. The remaining sources constitute the last group. While they do contain vulnerabilities affecting IoT products, they do not provide any classification of the affected products’ types, which would let one distinguish the IoT vulnerabilities from the others. The sources’ distinction into relevancy groups is shown in Table 10.

We did not find any source that would try to assign its vulnerabilities to the specific layer of the IoT paradigm. The only valuable observation in this regard is that, due to the nature of hacks presented in the exploitee.rs wiki, its entries can be considered as relating to the perception layer.

### 4.2. Number of Entries

This section compares the number of entries found in the sources. This metric can easily show the size of the source, but it can be misleading in some cases. For example, some sources, such as NVD, create a separate database entry for each vulnerability, while in other sources—CERT bulletins or blogs—A single entry often includes multiple vulnerabilities. We also presented one source that aggregates data from other sources, i.e., Vulners, and its number of entries is the highest, but in many cases, it has the same vulnerabilities described in multiple separate entries originating from different bulletins. Other sources, most notably US-CERT ICS Advisories, bind multiple vulnerabilities into a single entry, which reduces their number of entries. At this stage of our research, we do not have a reliable mechanism for determining whether a given entry is or is not IoT related, so we cannot yet present numbers representing the IoT itself. Another missing piece of information is the collective amount of entries from unstructured sources, as it cannot be reliably estimated. A comparison of the number of entries is presented in Table 11. Figures have been checked as of 7th September 2020, unless otherwise noted.

The highest amount of entries is found on Vulners, which aggregates multiple sources—Including NVD, US-CERT, ZDI and the English-language JVN entries. The general vulnerability databases and Bugtraq have a similar number of vulnerabilities, as they are cataloguing software vulnerabilities in a broad scope. The remaining sources include fewer entries, since their content is more concentrated. ZDI publishes only advisories on vulnerabilities reported through its vulnerability disclosure program, and other sources report only a selection of vulnerabilities affecting ICS or IoT devices.

### 4.3. CVE Use

Among the considered sources, only exploitee.rs does not reference CVE identifiers in any of its entries. This can be justified, as it focuses on hardware hacks and exploits rather than on the individual vulnerabilities. For the remaining sources the rate of CVE adoption varies, with the highest rate, 100%, observed for the NVD. While inclusion of the CVE identifiers is useful, the lower adoption rates do not have to be considered as a drawback. On the contrary, entries without CVE identifiers are likely to be describing original vulnerabilities not portrayed in the NVD and are therefore a valuable source of new information. The summary of CVE inclusion rates is presented in Table 12. Some sources are not presented in the table: exploitee.rs, due to not using CVE, Chinese ICS CERT, as its vulnerability descriptions are copied from either CNVD or CNNVD, and Vulners, as its CVE rate is dependent on the original sources’ rates. This analysis was performed on the 13 February 2020, so the numbers of entries found in sources are lower than these presented in the previous section.

The CNVD XML feed started in January 2015. To put its numbers into perspective: NVD published 71,071 entries since then, including 63,899 valid entries and 7172 rejected CVEs. It should be noted that the fact of not providing CVE ID for the entry in a given source is not enough to imply that the considered vulnerability did not receive its CVE. For example, there are CNVD vulnerabilities without CVE ID, but having Bugtraq ID. Using the latter, we were able to trace these vulnerabilities’ CVEs back. These entries: CNVD-2011-5107, CNVD-2011-5108, CNVD-2011-5110, CNVD-2011-5103 and CNVD-2011-5105 all reference BID:50828, which lists CVE-2011-4875, CVE-2011-4876 and CVE-2011-4877. Oddly, there are later CNVD entries that do mention these CVEs, but do not provide Bugtraq ID: CNVD-2012-0465, CNVD-2012-0466, and CNVD-2012-0467.

### 4.4. Vulnerability Scoring

The sources use vulnerability scoring to help their users prioritize the threats. Most sources use CVSS v2 [23] or CVSS v3 [24] vectors and scores, which are then used to determine the severity according to the rules shown in Table 8. One notable exception is CNNVD, which presents only a severity rating without CVSS score and vector, but, as shown in Section 3.3, these ratings are usually derived from scores published by the NVD. Some sources are not included in the table: Bugtraq and exploitee.rs do not provide any scores or severity evaluation, and for Vulners and ICS-CERT-CN the availability of this data is dependent on the original source. For each scoring system used by a given source, the adoption rate is calculated by authors of the paper on the basis of acquired data, by counting the number of entries that provide scores in a given system and the total number of entries available from the source. The adoption rate (AR) is defined as the ratio of the number of entries containing scores in a given system to the total number of entries available from the source. For example, for CVSS v3 in:

(1) $${\mathrm{AR}}_{\mathrm{CVSSv}3}=\frac{N_{\mathrm{CVSSv}3}}{N}\times 100\%,$$

where N is the total number of entries found in the source and N_CVSSv3_ is the number of entries with CVSS v3 scores provided.

As some sources use more than one scoring system, the calculation is done independently for each system employed by the source. In most sources using CVSS, the severity ratings are based strictly on the CVSS scores, but CNVD provides severities even for sources without CVSS scores, therefore for this source adoption rate for severity rating was also calculated independently. The summary of scoring systems’ usage is presented in Table 13.

Most sources seem to follow NVD in their selection of vulnerability scoring system—JVNDB has very similar percentages, US-CERT and ZDI also started using CVSS v3. ZDI is unique in this group, as it is the only one that dropped CVSS v2 completely in favour of v3 while other sources use both versions simultaneously. Chinese sources offer less scoring information—CNNVD shows only a severity rating, and CNVD does not use CVSS v3 yet. CNVD is also the only source that offers CVSS ratings but has less than 90% of its entries scored. Finally, there are two sources that provide no vulnerability scoring whatsoever, i.e., Bugtraq and exploitee.rs. Additional remarks supplementing the data presented in Table 13:

- NVD switched from CVSS v3.0 to CVSS v3.1 scoring on 10 September 2019 [71]. Most of the 0.2% entries without scores are new entries awaiting scoring.
- JVNDB started adding CVSS v3 vectors in 2016.
- JVNDB erroneously assigns CVSS v3.0 version to CVSS v3.1 vector obtained from the NVD.
- JVNDB provides the source of every CVSS score that it presents.
- Calculations for ICS-CERT should be treated as an estimate, as its advisories do not have a consistent data structure and sometimes include multiple vulnerabilities listed under the same ID.
- ZDI uses only one CVSS version for every entry, i.e., if CVSS v3 vector is provided, there is no CVSS v2 and vice versa. The CVSS v2 scores started being added in 2010 (only 3 out of 6706 advisories published since 2010 have no CVSS scoring), and the switch to the CVSS v3 happened gradually between late 2018 and early 2019.
- CNVD provides severity ratings for all vulnerabilities, even those without CVSS vectors. There is one exception found in the XML feed that does not have it, but it is a test entry with both its title and description containing only the word “test”.

### 4.5. Affected Products Identification

The last compared factor is the method of indicating what products are actually vulnerable. NIST released the CPE (common platform enumeration) [25] naming scheme to be used for this purpose. It is a structured naming scheme suitable for machine processing. However, the CPE is used by only a few sources—the NVD itself, JVNDB, and, to some extent, CNNVD. In CNNVD’s case, CPEs are only directly provided in the XML files. The affected products lists presented on the website are constructed using parts of the CPE identifiers, but the presented name formats are not consistent. For some entries the vendor name is separated from the product name using a space, the version number follows the product name after a colon and other parts of CPE are omitted, e.g., “Google Android:4.1.2”. Sometimes the colon between vendor and product names is kept, as well as the following colons after the version name, but without asterisks: “Paloaltonetworks:Pan-os:7.0.5:::”, while some other entries keep the asterisks too: “Openssl:Openssl:1.0.2c:*:*:*”. Only CNNVD entries referencing CVE ID have affected products lists, suggesting that CNNVD does not create its own CPEs but relies on these published by the NVD. Other sources do not use CPE at all, and indicate vulnerable products using an unstructured, human readable format, which hinders the ability of automatic data processing. The highest difficulties grow in regard to determining the affected products versions, which can be expressed in multiple ways. For example, US-CERT/ICS uses expressions like “all versions“, “all versions prior to” or “Versions 4.2 and prior”, and CNVD uses version descriptions like “v4.1.5”, “<2.1.0” or “≥13.0.0, ≤13.1.0.5”. Even within the same entry version numbers can be provided inconsistently: “V1.2.2.65” and “V1.2.2 build 64”. This makes determining vulnerable products and versions particularly challenging for vulnerabilities found only in these sources.

## 5. Discussions and Open Issues

As shown in the analysis of individual sources, a single, comprehensive, IoT-dedicated vulnerability database does not exist yet. The available solutions were created with software vulnerabilities in mind and are not well-suited to manage the vulnerabilities affecting the IoT world, in which multiple domains, e.g., hardware, software, and networking, cross over. Although we have managed to find some sources that have tried to focus on IoT, none of them are useful in solving the IoT security problem. All these sources are already defunct or no longer updated (such as secureplanet.io or exploitee.rs), and none of them have ever contained an extensive collection of vulnerabilities. Hence, one of the challenges lies in distilling the IoT vulnerabilities from the other sources. This task is easier for the ICS devices, for which dedicated sources already exist, such as US-CERT’s ICS bulletins or two Chinese sources, namely IVD and ICS-CERT-CN. For the consumer-grade IoT devices, we were able to identify only one dedicated source, exploitee.rs, but it covers only a small range of devices and no longer receives updates. Among the general vulnerability databases, only the CNVD offers a dedicated IoT category, but its execution is not perfect as it misses many IoT devices and IoT related software, but includes non-IoT devices (by the definition used in this paper) like smartphones. For other sources, the lists of vulnerabilities affecting the IoT have to be constructed by their users themselves. This cannot be easily done in an automatic way, as it would require prior knowledge about IoT vendors and product names. While there are some efforts in cataloguing the IoT devices, such as [72], their scope is too limited at the moment to prove any usability in this case.

One noteworthy finding is the fact that the largest sources beside Vulners and NVD, i.e., CNVD, CNNVD, and JVNDB provide CVE numbers for most of their entries. Moreover, the individual vulnerability data often matches those provided by NVD. This can be seen on the CNNVD severity chart in Section 3.3, or by the directly provided CVSS source in JVNDB, which often states NVD as the source. This limits the usability of these sources, as large part of their data is already available in NVD without the need to translate them to English. Only CNVD contains a relatively large number of non-CVE vulnerabilities (about 20% of all its entries). Even for CNVD entries with CVE numbers, the CVSS vectors often differ from those found in NVD, suggesting that CNVD scores vulnerabilities based on its own analysis.

Other sources can play only a supplementary role. CERT bulletins and ZDI advisories cover less vulnerabilities than NVD and use data formats less suitable for machine processing, but often provide information about vulnerabilities before they are published in NVD. Bugtraq has not been receiving updates since July 2019, and Vulners only increases convenience by aggregating entries from multiple sources in one service.

Since many vulnerabilities and exploits affecting IoT devices are never catalogued in databases, additional sources have to be browsed to keep awareness in the IoT threat landscape. Good sources are blogs, but it is hard to track and extract relevant information from them. They are written by either the individual researchers, hacker groups, or security companies, presenting their accomplishments in smart device hacking. As they are rarely dedicated solely to this topic, individual IoT related posts have to be filtered out. Moreover, as new blogs are created and older ones stop posting new articles, a crawling mechanism actively searching the web for new sources is needed to keep the incoming data streams alive.

## 6. Conclusions and Future Work

As the usage of IoT products over the wide range of applications is growing rapidly, their security becomes an increasingly growing concern. A large number of vulnerabilities coupled with lacking product support and patching processes pose a threat to the economy, citizens’ security, and privacy. Insecure IoT devices are already exploited and used in massive attacks, which may become even larger and more frequent if no action is taken to secure the IoT environment. Solving these issues would greatly benefit from the existence of a publicly available source of structured information about known IoT vulnerabilities and exploits. Currently, none of the existing solutions is satisfactory, which highlights the need for the database focused on the IoT. Creation of such a database is one of the goals of the Vulnerability and Attack Repository for IoT (VARIoT) project [73]. The main contribution of the paper is setting the ground for this task by in-depth evaluating available sources of information about vulnerabilities of IoT. The second contribution is the indication of shortcomings of these sources in relation to the IoT paradigm. General requirements for a comprehensive IoT database are also pointed out in this paper, which can also be perceived as an important outcome.

The sources identified in this paper will be used in future work under the VARIoT project. Our goal is to mitigate the issue of unsatisfactory IoT vulnerability data availability by creating an IoT-oriented database, which will contain information gathered from the sources identified during this research. The data will be processed by correlating information from different sources and enriched with information about affected products provided in a way suitable for the needs of the IoT environment, for example by adding information about the IoT layer affected by the vulnerability, which is missing from the source data. The data will be publicly available and presented in a format that can be processed both manually and in an automatic way by various types of security software. This will lead to an increase in the cybersecurity of entities using this database and, consequently, the entire IoT world.

## Figures and Tables

**Table 1:** A short overview of the sources discussed in this work.

Section	Name	Description
3.1	NVD	American National Vulnerability Database, the de facto standard vulnerability information source in the IT-sec industry
3.2	CNVD	China National Vulnerability Database, a similar database maintained by the Chinese national computer emergency response team (CERT). Often presents vulnerabilities unavailable in other sources
3.3	CNNVD	Chinese National Vulnerability Database of Information Security, a second database from China. Usually follows data found in NVD
3.4	JVNDB	Japan Vulnerability Notes iPedia, Japanese NVD counterpart. Mostly follows NVD but contains some additional entries
3.5	IVD	ICS Vulnerability Database from a Chinese ICS security company Winicssec. Contains data from other sources (NVD, CNVD and CNNVD)
3.6	ICS-CERT-CN	Chinese national CERT’s ICS branch, whose website contains a list of ICS and IoT vulnerabilities. These vulnerabilities are found in either CNVD or CNNVD
3.7	US-CERT	US-CERTs ICS advisories. Unlike these from Chinese CERT, they are original entries whose publication usually precedes publications in databases
3.8	Other CERTs	Other CERTs’ websites, which were determined as less useful and hence grouped together in a single entry
3.9	ZDI	Website of Zero Day Initiative bug bounty program with advisories on found vulnerabilities, which often precede publications in databases
3.10	Bugtraq	Independent list of vulnerabilities. No longer updated but contains a considerable amount of archival information
3.11	Vulners	An extensive database aggregating vulnerability and exploit data from over 130 sources
3.12	Exploitee.rs	Small wiki dedicated to hacking consumer-grade IoT devices
3.13 and 3.14	Other	Other sources bearing less significance. Divided in two categories: structured sources (3.13) present data in a structured way, e.g., have lists of vulnerabilities, database-like fields etc. Unstructured sources provide only human readable data, e.g., as writeups on blogs

**Table 2:** Data fields presented in the NVD entries.

Field Name	Availability in JSON Feed	Description
CVE Dictionary Entry	yes	CVE ID
NVD Published Date	yes	Date of publication in NVD
NVD Last Modified	yes	Date of last data modification in NVD
Source	yes	CVE source, always states “MITRE”
Current Description	yes	Description of the vulnerability
Analysis Description	no	Usually the same as the current description
Severity	yes 1	CVSS scores and severity metrics resulting from them
References	yes	Links to external sources of information
Weakness Enumeration	yes 1	Common Weakness Enumeration (CWE) ID associated with a vulnerability
Known Affected Software Configurations	yes 2	List of vulnerable CPE configurations
Change History	no	History of changes made to the entry since its publication

^1^ CVSS scores and weakness enumeration fields available in the JSON files do not include information about source, and do not include CVSS scores from CNAs if they do not match with NVD scores. ^2^ CPE versions 2.3 and 2.2 are both available on the website, JSON feed contains only CPE 2.3.

**Table 3:** List of CNVD vulnerability categories and subpages. Vulnerability counts presented as of 25th August 2020. Own work based on [27].

Subpage or Category Name	Type	Vulnerability Count
Telecommunications	subpage	7536
Mobile Internet	subpage	9214
ICS	subpage	2728
WEB Application	category	25,022
Blockchain Public Chain	category	356
Blockchain Consortium Chain	category	2
Security Product	category	2185
Application	category	70,130
Operating System	category	12,205
Database	category	2305
Smart Devices (IoT terminal devices)	category	504
Network Equipment (network devices such as switches and routers)	category	7683
Vulnerability list (all vulnerabilities)	-	146,342

**Table 4:** Characterization of the data fields available in the CNVD entries found on the website and in the XML feed.

Data Field	XML Availability	Description
Title	yes	Vulnerability title, including vulnerable product name and vulnerability type
CNVD-ID	yes	ID within the CNVD database
Publication date	yes	Date of publication in CNVD
Severity	partial ^1^	CNVD v2 vector and severity rating based on it
Affected products	yes	List of vulnerable products
CVE ID	yes	Related CVE IDs
Bugtraq ID	yes	Related BIDs
Other ID	no	IDs from other sources, such as OSVDB or exploit-db
Description	yes	Vulnerability description
Vulnerability type	yes	This field contents are the same for all reviewed vulnerabilities, stating “generic vulnerability”
References	yes	Links to external sources of information on the vulnerability
Solution	yes	Information about possible vulnerability remediation and vendor patches
Vendor Patch	no	Link to CNVD patch entry for a given vulnerability. These entries contain short patch descriptions and links to download pages. Some patches can be also downloaded straight from the CNVD
Verification	no	Vulnerability verification by CNVD. Can have one of two values: “verified” or “no verification information”
Update date	no	Date of the latest entry update
Inclusion date	no	Date of adding entry to the database. Can be earlier than publication date
Submission date	yes	Date of receiving first information about the vulnerability
Attachment	no	Files attached to the entry. In most cases either no attachment exists or attachment is not made publicly available

^1^ XML feed contains only the severity rating (LOW, MEDIUM or HIGH) without CVSS vectors. The severity field label in feed files is misspelled “serverity”.

**Table 5:** Number of referenced CVE IDs per CNVD entry.

CVE ID Count in a Single CNVD Entry	Number of CNVD Entries	Percentage of CNVD Entries
0	2582	22.6%
1	8819	77.2%
2	13	0.1%
3 or more	7	0.1%
Total	11,421	100%

**Table 6:** CNVD and NVD CVSS vectors compatibility, checked for a subset of IoT-related CNVD entries that include CVSS scores and CVE IDs.

Quantity	Count	Percentage
Matching CVSS vectors	4828	59.1%
Differing CVSS vectors	3276	40.1%
CVE not found in NVD	42	0.5%
CVSS vectors missing in NVD	23	0.3%
CNVD entries including both CVE and CVSS	8169	100%

**Table 7:** Description of data fields available in CNNVD entries on the database webpage.

Data Field	NVD Counterpart	Description
Title	-	Vulnerable product name and type of vulnerability
CNNVD ID	-	entry ID within the CNNVD database
CVE ID	CVE Dictionary Entry	Related CVE ID, at most one per entry
Release date	NVD Published Date	Date of publication in the database
Update date	NVD Last Modified	Date of last entry modification
Source	-	Person or organization reporting the vulnerability. Different than the NVD “Source” field
Severity	Severity	One-word vulnerability severity rating
Vulnerability type	CWE	Type of vulnerability, similar to CWE values
Threat type	CVSS AV metric	Simplified Attack Vector metric—Can take one of two values, “remote” or “local”
Manufacturer	-	Vendor of the vulnerable product
Description	Current Description	Short description of the vulnerability
Solution	-	Information about remediations, including vendor patches
References	References	Links to external sources
Affected products	Configurations	List of vulnerable products
Patch	-	Links to CNNVD subpages with further patch information. Each entry can include more than one link

**Table 8:** Calculation of the severity ratings based on the CVSS scores, according to [33].

CVSS v2.0 Ratings		CVSS v3.0 Ratings	
Severity	Base Score Range	Severity	Base Score Range
		None	0.0
Low	0.0–3.9	Low	0.1–3.9
Medium	4.0–6.9	Medium	4.0–6.9
High	7.0–10.0	High	7.0–8.9
		Critical	9.0–10.0

**Table 9:** Description of data fields available in the JVNDB entries.

Field Name	Description
Title	Vulnerability title, contains the vulnerable product name and vulnerability type
Overview	Short description of the vulnerability
CVSS Severity	CVSS v2 and CVSS v3 vectors and scores. If there are multiple CVEs per entry, only one set of CVSS scores is available from the data feed, but all scores can be accessed on the website. Each score contains the information identifying the authority issuing the score—Usually IPA or NVD
Affected Item	List of vulnerable products. On the website this field can contain some additional information unavailable in the XML feed
Expected Impact	Possible harm due to successful exploitation. Field unavailable in the XML feed
Solution	Patches, countermeasures and workarounds that can mitigate the vulnerability
Vendor Information	Advisories, statements etc. issued by vendors of vulnerable products
CWE	Vulnerability type according to CWE list
CVE	List of all CVE IDs related to the entry
References	Links to external sources of information
Revision History	History of changes to the entry. Unlike NVD, which shows revision history only on the website, JVNDB also includes it in the XML data feed
Publication Date	Date when the vulnerability became publicly known
Registration Date	Date when the JVNDB entry was created
Last Updated	Date of the last entry update
CPE	CPE identifier of the vulnerable configuration. Uses CPE 2.2 and is only available in the XML feed

**Table 10:** Data sources grouped by their relevancy for IoT.

Relevancy Group	Sources
1. IoT or ICS focused	Exploitee.rs, IVD, ICS-CERT-CN, US-CERT/ICS
2. Has some IoT amenities	CNVD
3. Not focused on IoT	NVD, CNNVD, JVNDB, ZDI, Bugtraq, Vulners

**Table 11:** Comparison of the number of entries found in each source.

Source	Number of Entries	Comments
NVD	149,261	-
CNVD	147,204	-
CNNVD	150,236	-
JVNDB	121,388	Checked at 13 July 2020
IVD	3454	-
ICS-CERT-CN	3627	-
US-CERT	1443	Only ICS-CERT advisories counted. Many entries describe multiple vulnerabilities each
ZDI	7614	Checked at 13 July 2020
Bugtraq	100,825	-
Vulners	1,847,228	-
exploitee.rs	60	-

**Table 12:** CVE inclusion rate—Ratio of the number of entries that include CVE IDs to the total number of entries available in the database.

Database	Entries with CVE	All Entries	Percentage
NVD	138,698	138,698	100.0%
CNNVD	131,464	139,708	94.1%
CNVD (XML Feed)	52,423	64,366	81.4%
CNVD (IoT Related)	7491	9806	76.4%
JVNDB	113,599	113,999	99.6%
IVD	2417	3136	77.1%
ZDI	6049	7040	85.9%
Bugtraq	73,772	100,825	73.2%
CERT ICS	1268	1315	96.4%

**Table 13:** Vulnerability scoring systems and their adoption rates across the sources. The percentages for severity are omitted in most cases, since this rating is derived from the CVSS score. The exception is made for CNNVD, which does not show CVSS scores and CNVD, which shows severity even for sources without CVSS scoring.

Database	Scoring System	Adoption Rate	Comments
NVD	CVSS v2	99.8%	-
	CVSS v3	43.6%	99.4% since 2017
CNNVD	Severity	93.9%	-
CNVD (XML feed)	Severity	100.0%	-
CNVD (IoT related)	CVSS v2	85.7%	-
	Severity	100.0%	-
JVNDB	CVSS v2	99.8%	-
	CVSS v3	47.8%	99.8% since 2017
IVD	CVSS v2	94.0%	-
ZDI	CVSS v2	74.7%	96.4% from 2010 up to 2018
	CVSS v3	20.5%	95.4% since 2019
	CVSS (any version)	95.2%	99.96% since 2010
US-CERT ICS	CVSS v2	92.5%	Calculated per advisory
	CVSS v3	65.2%	Calculated per advisory
